# Modification of serum fatty acids in preterm infants by parenteral lipids and enteral docosahexaenoic acid/arachidonic acid: A secondary analysis of the Mega Donna Mega trial

**DOI:** 10.1016/j.clnu.2023.04.020

**Published:** 2023-04-17

**Authors:** Ulrika Sjöbom, Mats X. Andersson, Aldina Pivodic, Anna-My Lund, Mireille Vanpee, Ingrid Hansen-Pupp, David Ley, Dirk Wackernagel, Karin Sävman, Lois E.H. Smith, Chatarina Löfqvist, Ann Hellström, Anders K. Nilsson

**Affiliations:** a Section for Ophthalmology, Department of Clinical Neuroscience, Institute of Neuroscience and Physiology, Sahlgrenska Academy, University of Gothenburg, Gothenburg, Sweden; b Learning and Leadership for Health Care Professionals at the Institute of Health and Care Science at Sahlgrenska Academy at University of Gothenburg, Gothenburg, Sweden; c Department of Biological and Environmental Sciences, University of Gothenburg, Gothenburg, Sweden; d Lund University, Skåne University Hospital, Department of Clinical Sciences Lund, Pediatrics, Lund, Sweden; e Department of Women’s and Children’s Health, Karolinska Institutet and Karolinska University Hospital, Stockholm, Sweden; f Department of Neonatology, Karolinska University Hospital and Institute, Astrid Lindgrens Children’s Hospital, Stockholm, Sweden; g Department of Pediatrics, Institute of Clinical Sciences, Sahlgrenska Academy, University of Gothenburg, Gothenburg, Sweden; h Region Västra Götaland, Department of Neonatology, The Queen Silvia Children’s Hospital, Sahlgrenska University Hospital, Gothenburg, Sweden; i The Department of Ophthalmology, Boston Children’s Hospital, Harvard Medical School, Boston, MA, USA

**Keywords:** Arachidonic acid, Docosahexaenoic acid, Extremely preterm infants, Intravenous lipid emulsion, LCPUFA, Parenteral nutrition

## Abstract

**Background & aim:**

Preterm infants risk deficits of long-chain polyunsaturated fatty acids (LCPUFAs) that may contribute to morbidities and hamper neurodevelopment. We aimed to determine longitudinal serum fatty acid profiles in preterm infants and how the profiles are affected by enteral and parenteral lipid sources.

**Methods:**

Cohort study analyzing fatty acid data from the Mega Donna Mega study, a randomized control trial with infants born <28 weeks of gestation (n = 204) receiving standard nutrition or daily enteral lipid supplementation with arachidonic acid (AA):docosahexaenoic acid (DHA) (100:50 mg/kg/day). Infants received an intravenous lipid emulsion containing olive oil:soybean oil (4:1). Infants were followed from birth to postmenstrual age 40 weeks. Levels of 31 different fatty acids from serum phospholipids were determined by GCeMS and reported in relative (mol%) and absolute concentration (μmol l^−1^) units.

**Results:**

Higher parenteral lipid administration resulted in lower serum proportion of AA and DHA relative to other fatty acids during the first 13 weeks of life (p < 0.001 for the 25th vs the 75th percentile). The enteral AA:DHA supplement increased the target fatty acids with little impact on other fatty acids. The absolute concentration of total phospholipid fatty acids changed rapidly in the first weeks of life, peaking at day 3, median (Q1-Q3) 4452 (3645–5466) μmol l^−1^, and was positively correlated to the intake of parenteral lipids. Overall, infants displayed common fatty acid trajectories over the study period. However, remarkable differences in fatty acid patterns were observed depending on whether levels were expressed in relative or absolute units. For example, the relative levels of many LCPUFAs, including DHA and AA, declined rapidly after birth while their absolute concentrations increased in the first week of life. For DHA, absolute levels were significantly higher compared to cord blood from day 1 until postnatal week 16 (p < 0.001). For AA, absolute postnatal levels were lower compared to cord blood from week 4 throughout the study period (p < 0.05).

**Conclusions:**

Our data show that parenteral lipids aggravate the postnatal loss of LCPUFAs seen in preterm infants and that serum AA available for accretion is below that *in utero*. Further research is needed to establish optimal postnatal fatty acid supplementation and profiles in extremely preterm infants to promote development and long-term health.

## 1. Introduction

Extremely preterm infants (born <28 weeks’ gestation) often show low postnatal levels of long-chain polyunsaturated fatty acids (LCPUFA) associated with an increased risk for neonatal morbidities [1–5]. Preterm infants can synthesize the omega-6 LCPUFA arachidonic acid (AA, 20:4 n–6) and the omega-3 LCPUFA docosahexaenoic acid (DHA, 22:6 n–3) from the precursors linoleic acid (LA 18:2 n–6) and α-linolenic acid (ALA, 18:3 n–3), respectively [6–9]. However, although the capacity of endogenous synthesis is significant, estimates of accretion rates suggest that supplementation with AA and DHA is required to match the infant’s needs [10].

Fortification of intravenous lipid emulsions and enteral supplementation have been assessed as ways to improve LCPUFA status in preterm infants [11–19]. Supplementation has primarily focused on DHA, but DHA together with AA, has also been evaluated [12,16,18–20]. Fish oil-containing intravenous lipid emulsions, now widely used in neonatal intensive care units, have a high DHA and eicosapentaenoic acid (EPA, 20:5 n–3) content but are relatively low in AA [21]. Consequently, these emulsions increase infant DHA and EPA but reduce their AA levels, resulting in an AA:DHA ratio that may be non-physiological [11,14,17]. Enteral supplementation with fish oil or human milk with high DHA concentration has a similar effect on circulating LCPUFA levels in the preterm infant, i.e. DHA levels increase while AA is reduced [13,15]. A limitation of many previous studies assessing LCPUFA status in preterm infants is that only a few longitudinal samples have been evaluated for fatty acid status. Therefore, changes in blood fatty acid composition related to nutrition and metabolism may go undetected.

The Mega Donna Mega (MDM) trial recently investigated the effect of enteral supplementation with AA:DHA (2:1 ratio) on the outcome of severe retinopathy of prematurity (ROP) in extremely preterm infants. Daily supplementation from birth to term equivalent age significantly reduced the risk for severe ROP by 50% and increased the molar fractions of AA and DHA in serum phospholipids [12].

In this secondary analysis of the MDM trial, we aimed to investigate infants’ serum complete fatty acid profiles over the first months of life, expressed in relative and absolute units, and how the profiles were modulated by the administration of parenteral lipids and enteral AA:DHA supplementation.

## 2. Materials and methods

### 2.1. Study design

The current study was performed within the randomized, open-labeled Mega Donna Mega trial [12]. Postnatal serum fatty acid composition was a pre-defined secondary outcome of the trial. The study protocol can be found at ClinicalTrials.gov (Identifier: NCT03201588).

### 2.2. Participants and intervention

The Regional Ethical Review Board at the University of Gothenburg approved the study (Dnr 303–11, T570–15). The study was performed following theethical standards of the Helsinki Declaration of 1975, as revised in 1983. Informed signed consent for all participants was obtained from parents or legal guardians. Infants were recruited between December 2016 and August 2019 at three centers in Sweden: Queen Silvia Children’s Hospital in Göteborg, Skåne University Hospital in Lund, and Karolinska University Hospital in Stockholm [12]. The randomization strategy and inclusion/exclusion criteria have been previously described [12]. The intervention supplement was Formulaid^™^ (DSM Nutritional Products, Heerlen, Netherlands), a triglyceride oil made from extracts of the filamentous fungus *Mortierella alpina,* and the dinoflagellate *Crypthecodinium cohnii,* containing AA and DHA in a two-to-one ratio. Formulaid^™^ was administrated enterally starting within 72 h after birth and lasted until 40 weeks postmenstrual age (PMA). The dose was 0.39 mL kg^−1^ daily, equal to 100 mg AA and 50 mg DHA per kg body weight and day. Infants assigned to control received standard care.

### 2.3. Collection of clinical data

Nutritional-, growth-, morbidity-, and mortality data were collected according to the study protocol, following infants from birth to 40 weeks PMA [12]. Daily data on the parenteral and enteral administration was registered in the Nutrium^™^ Software (Nutrium AB, Umeå, Sweden). Standardized birth weight was calculated based on growth charts by Fenton and Kim [22].

### 2.4. Nutritional practices

Details of the nutritional strategy have been described [17,23]. The parenteral nutrition was initiated as early as possible after birth and was provided either via 3-in-1 (Numeta G13E, Baxter Medical AB, Kista, Sweden) or by individually composed parenteral nutrition consisting of glucose (monohydrate 50 g 100 ml^−1^), amino acids (2 g 100 ml^−1^ Vaminolac^®^, Fresenius Kabi, Uppsala, Sweden or 3.1 g 100 ml^−1^ Primene, Baxter Medical AB, Kista, Sweden), and lipids. The composite lipid emulsion Clinoleic 200 mg ml^−1^ (Baxter Medical AB, Kista, Sweden), based on olive oil (80%) and soybean oil (20%), was used at all sites either as part of Numeta G13E or as part of individually composed parenteral nutrition. Parenteral and concomitant enteral lipid, protein and carbohydrate intakes were gradually increased during the following 3–4 postnatal days according to Swedish national guidelines targeting a lipid intake of 3–4 g/kg/d, protein of 3.5–4 g/kg/d, and carbohydrate of 11–16 g/kg/d. The parenteral nutrition was supplemented with fat-soluble vitamins (Vitalipid^®^, Fresenius Kabi), water-soluble vitamins (Soluvit^®^, Fresenius Kabi), and trace elements (Peditrace^®^, Fresenius Kabi). Enteral feeds were introduced, if possible, from the first day of life and consisted of mothers’ own milk, if available, and otherwise of pasteurized donor human milk. Enteral feeds were advanced according to the infant’s feeding tolerance to a target volume of 160–180 ml kg^−1^ day^−1^. Individualized targeted fortification, based on weekly macronutrient analyses of either mother’s own or donor milk, was done using human milk fortifiers (Nutriprem HMF, Nutricia; PreNAN HMF, Nestlé; or Enfamil HMF, Mead Johnson) and/or fat emulsions and/or carbohydrates. Donor milk was replaced by preterm formula beyond 34 weeks PMA.

### 2.5. Blood sample collection and fatty acid analysis

Blood was collected in serum separator tubes from cord blood (2 mL) and infant arterial or venous blood (0.6 mL) at postnatal age (PNA) 1, 3, 7, 14, 28, and 42 days and at postmenstrual age (PMA) 30, 32, 34, 36, and 40 weeks. Blood samples were allowed to clot for a minimum of 0.5 h and a maximum of 2 h before centrifugation (1500 *g* for 10 min), followed by serum collection. Samples were stored at −20 °C for up to one week before long-term storage at −80 °C.

For analysis, serum (25 mL) was spiked with an internal standard (1,2-dinonadecanoyl-sn-glycero-3-phosphocholine), and the sample was lyophilized. Total lipids were extracted [24], and a phospholipid fraction was obtained by fractionation on a SEP-PAK aminopropyl cartridge (Waters Corp.). Phospholipid-bound fatty acids were converted into fatty acid methyl esters (FAME) by acid-catalyzed transmethylation and analyzed by GC–MS as previously described [25]. Samples were assayed in 94 separate batches between September 2017 and December 2019. All samples from an individual were assayed in the same batch. An internal control sample consisting of pooled adult serum was included in each batch to follow variability in the method. Thirty-one fatty acids were quantified in all samples. For quantification, the ratios between the peak area of the internal standard (19:0 methyl ester) and the peak areas of each target fatty acid were compared to linear calibration curves made from authentic standards. Fatty acid concentrations were calculated in relative (mol%) and absolute serum concentration (μmol l^−1^) units.

We observed differences between extraction batches in the absolute quantification related to the analyst and the methylation reagent lot. The internal control sample was therefore used to normalize between extraction batches. Adjustments were made for each fatty acid separately, using a ratio between the concentration of the internal control in each batch and the mean concentration of the internal control sample for all batches. The inter-assay CV for the relative quantification of DHA, AA, and LA were 11.9%, 6.9%, and 6.8%, respectively. The inter-assay CV for the absolute quantification of DHA, AA, and LA were 10.2%, 9.2%, and 10.1%, respectively.

### 2.6. Statistical analyses

We previously [12] reported the relative serum levels of AA and DHA in infants based on a timescale that combined infants’ PNA with PMA. In this secondary analysis, data were analyzed based only on the infants’ PNA (Fig. S1). The rationale for using only the PNA-based time scale was to achieve a comparable exposure time of the enteral supplementation independently of GA at birth.

Infants were classified into three categories based on the administration of parenteral lipids during the first four weeks of life. Per each infant and day, the amount of administered parenteral lipids was standardized into a parenteral lipid standard deviation score (PNSDS) using the studied population’s daily means and standard deviations. Daily PNSDS were then flagged with higher intake if they were greater than 0, and the percentage of follow-up time on this higher intake was calculated (% time with PNSDS>0). Finally, % time with PNSDS>0 was categorized into three categories for analyses: ≤25th percentile, 25th-75th percentile, and ≥75th percentile. The amounts of administered parenteral lipids and their relationship to serum phospholipids were then described for the three categories.

Statistical analyses were performed using IBM SPSS Statistics version 27 (IBM Corp, Armonk, NY, USA) and SAS software version 9.4 (SAS Institute Inc., Cary, NC, USA). Rstudio Version 1.2.5033, R Version 4.0.4 [26], and the ggplot2 package was used for plotting results. For heatmaps, data was standardized with a standard deviation of 1 centered at 0, means were calculated for each time point, and plots were generated using the R package pheatmap. Identification of fatty acid profiles by clustering was performed as previously described [27]. UMAP transformation was done with the R package Uniform Manifold Approximation and Projection in R using default settings.

For differences in patient characteristic distribution between the two randomized groups, Fisher’s exact test was used for dichotomous variables and Mann–Whitney U-test for continuous variables. Test for trend between three categories of parenteral lipids was performed using Mantel–Haenszel Chi-Square test for dichotomous variables, Chi-Square test for non-ordered categorical variables, and Jonckheere–Terpstra test for continuous variables. Unadjusted pairwise Wilcoxon signed-rank test was used to compare fatty acid levels in cord blood with later postnatal time points.

Longitudinal fatty acids were analyzed using mixed models for repeated measures data with normal distribution, adjusting for within-infant correlation, and modeling age in days with the spline function. Fixed knots at 0.02, 0.05, 0.1, 0.3, and 0.5 fractions of time resulted in lower Akaike’s Information Criterion. The models included treatment arm, age (spline function), and interaction between intervention and age, and were adjusted for center, gestational age at birth, birth weight including interaction with age, and sex. Diagnostic plots of residuals were reviewed and found satisfactory. The difference between the estimated mean values of fatty acids for the two treatment arms was computed, adjusting the confidence intervals (CIs) and p-values for multiple testing using the Scheffe-Holm method. Similar models adjusted for GA at birth, birth weight, and both variables, including interaction with PNA and sex, were also performed for the three categories of PNSDS (≤25th, 25th-75th, ≥75th percentile). Statistical analyses were limited to the first 13 weeks to have a similar distribution of gestational ages at birth across the analysis period. All tests were two-sided and evaluated at a 0.05 significance level.

## 3. Results

### 3.1. Clinical characteristics of the study cohort

**Fig. 1.**
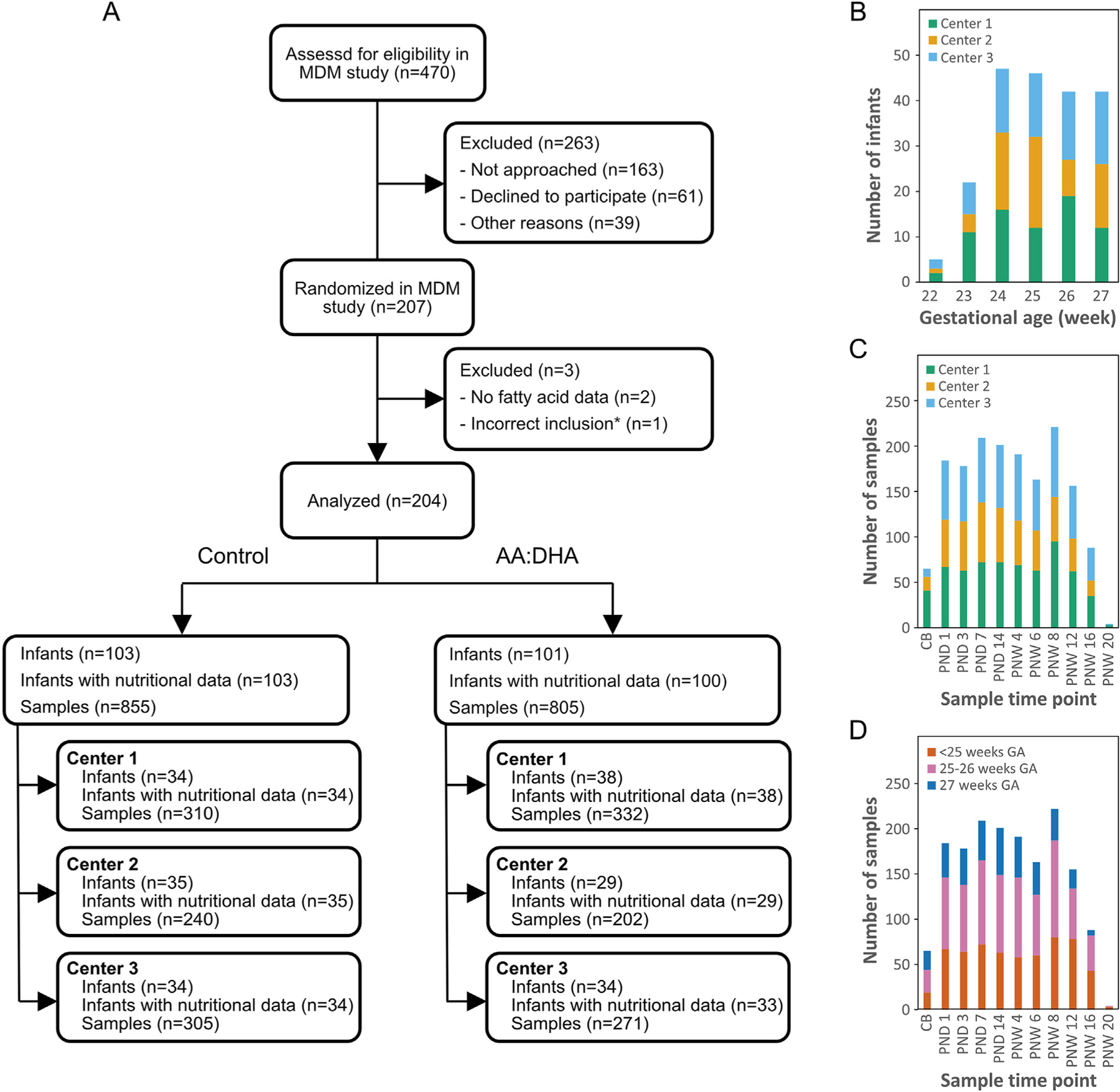
Cohort overview. **A** Flowchart of the cohort. **B** Number of infants included in the study by gestational age at birth and study center. **C** Number of serum samples used for fatty acid analysis according to sampling time and center. **D** Number of serum samples used for fatty acid analysis according to sampling time and gestational age stratification group. *malformation detected after randomization. PND, postnatal days; PNW, postnatal weeks.

An overview of the study cohort and samples used for fatty acid analysis is shown in Fig. 1A–D. Baseline characteristics were similar in infants receiving the AA:DHA supplement and infants receiving standard care (Table 1). The GA distribution was similar between centers (Fig. 1B). Cord blood samples were collected only from 65 infants, most of whom were cared for at center 1 (Fig. 1C). Samples collected at the latest postnatal time points were mainly from infants born at lower GAs, a consequence of the sampling scheme (Fig. 1D and Fig. S1).

### 3.2. Relation between parenteral lipids and serum phospholipids

Total phospholipid fatty acids increased rapidly from cord blood until postnatal day 3 (median [Q1-Q3] 2238 [1885–2696] μmol l^−1^ in cord blood and 4452 [3645–5466] μmol l^−1^ at day 3, p < 0.0001). The peak in serum phospholipid fatty acids around day 3 coincided with the maximum administration of parenteral lipids (Fig. S2).

**Fig. 2.**
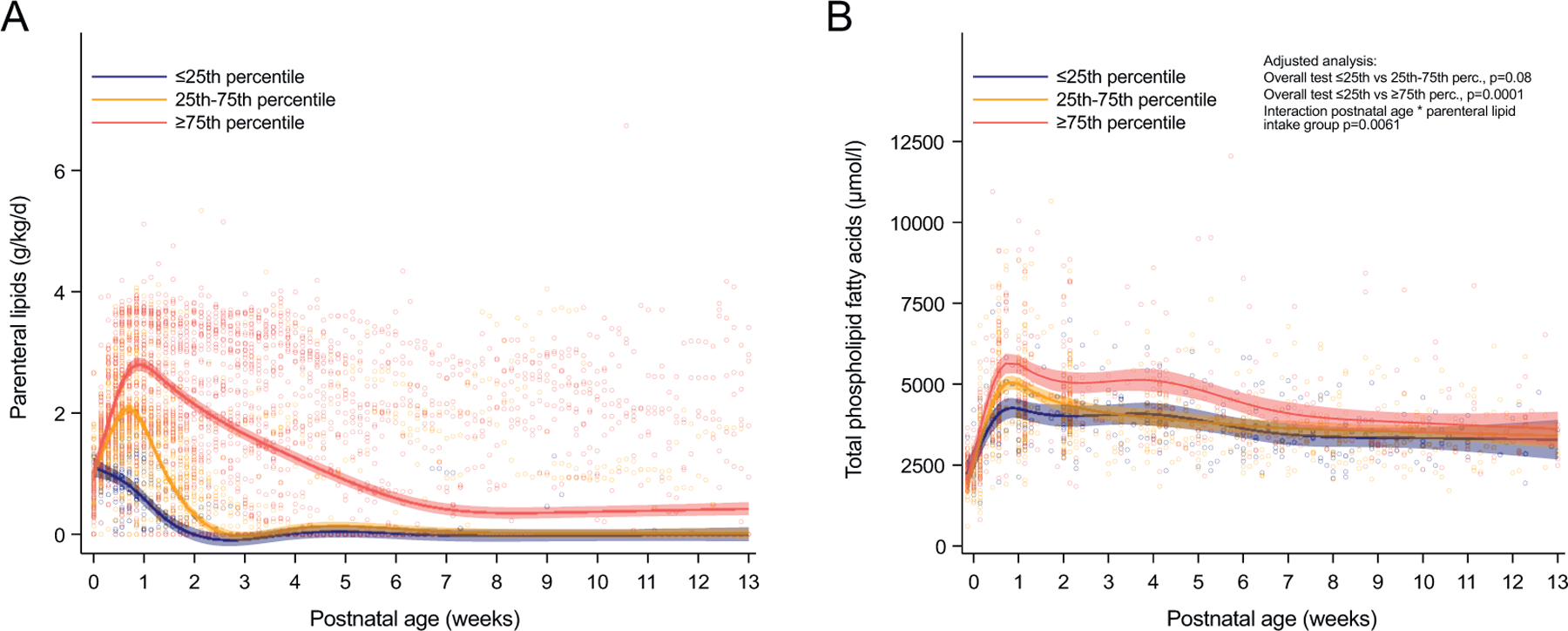
Relationship between serum phospholipids and administration of parenteral lipids. Infants are categorized into three groups according to their parenteral lipid intake in the first four weeks of life. (**A**) Daily infant intake of parenteral lipids over postnatal age. (**B**) Infant total serum phospholipid fatty acids over postnatal age. Lines represent estimates (with 95% CI) from a mixed model for repeated measures adjusted for GA at birth, birth weight, and both variables, including interaction with postnatal age and sex. N = 48 for ≤25th percentile, n = 102 for 25th-75th percentile, and n = 53 for ≥75th percentile.

To further investigate the relationship between serum fatty acids and parenteral lipids, infants were grouped into three categories based on percentiles of their standardized daily parenteral lipid intake in the first four weeks of life (Fig. 2A and Table 2). Infants with higher parenteral lipid intake had lower GA, birth weight, and birth weight SDS (Table 2).

Center 2 administered more parenteral lipids than the other two centers, resulting in more infants in the ≥75th percentile category and fewer infants in the ≤25th percentile category (Table 2). Infants in the ≥75th percentile category had significantly higher serum phospholipid fatty acids than infants in the ≤25th percentile category (Fig. 2B), with the most considerable difference seen around postnatal day 7.

### 3.3. Effect of parenteral lipids on serum AA and DHA

**Fig. 3.**
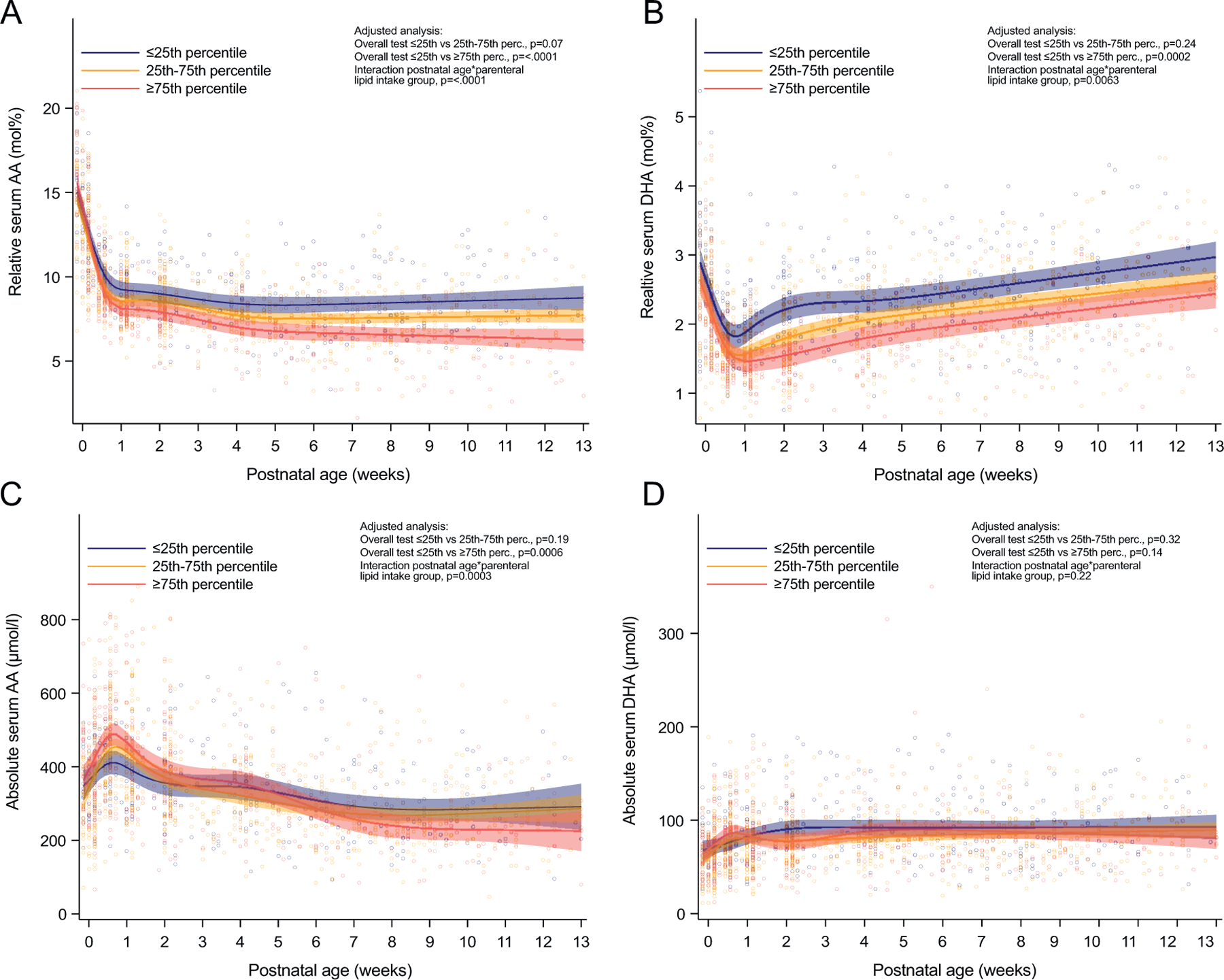
Relationship between parenteral lipids and infant serum AA and DHA levels. Infants are categorized into three groups according to their parenteral lipid intake in the first four weeks of life. Shown are relative levels (mol%) of AA (**A**) and DHA (**B**) and absolute levels (μmol l^−1^) of AA (**C**) and DHA (**D**). Lines represent estimates (with 95% CI) from a mixed model for repeated measures adjusted for GA at birth, birth weight, and both variables, including interaction with postnatal age and sex. N = 48 for ≤25th percentile, n = 102 for 25th-75th percentile, and n = 53 for ≥75th percentile.

Over the first 13 postnatal weeks, infants with the highest intake of parenteral lipids (≥75th percentile) showed significantly lower relative serum levels of AA and DHA compared to infants with the lowest intake (≤25th percentile) (Fig. 3A and B). By contrast, high vs low parenteral lipid intake increased absolute serum AA in the first week of life, whereas beyond postnatal week 7, absolute AA levels were suppressed (Fig. 3C). On day 3, the median (Q1-Q3) AA level in infants in the ≥75th percentile vs. the ≤25th percentile category was 486 (382–561) μmol l^−1^ vs. 427 (345–468) μmol l^−1^ (p = 0.022). No specific trends could be observed for absolute levels of DHA (Fig. 3D).

### 3.4. Enteral AA:DHA supplementation increases target fatty acids in infant serum

As we previously reported, infants who received the enteral AA:DHA supplement displayed higher relative levels of AA and DHA (Fig. S3 A and B). Infants in the intervention group also showed higher absolute levels of AA between weeks 9 and 11 (Fig. S3 C). Absolute serum DHA levels tended to be higher after supplementation but did not reach significance when adjusted for GA at birth, center, birth weight, postnatal age, and sex (Fig. S3 D). The AA:DHA intervention had minimal effects on the other measured phospholipid fatty acids (Fig. S4 [mol%] and S5 [μmol l^−1^]). In both treated and control groups, oleic (18:1 n–9) and linoleic acid were the fatty acids that showed the greatest change over time, both in relative and absolute units. The change in oleic acid was transient over the first week, whereas linoleic acid levels increased rapidly after birth and then were maintained at high levels throughout the study period. As no placebo was given to the control group, the additional fat provided to the intervention group through the supplement could have affected the total lipid intake. However, there was no difference between groups in lipid intake, nor in parenteral fluid or human milk intake over the first four weeks of life (Table S1).

### 3.5. Absolute and relative serum fatty acid levels over time

**Fig. 4.**
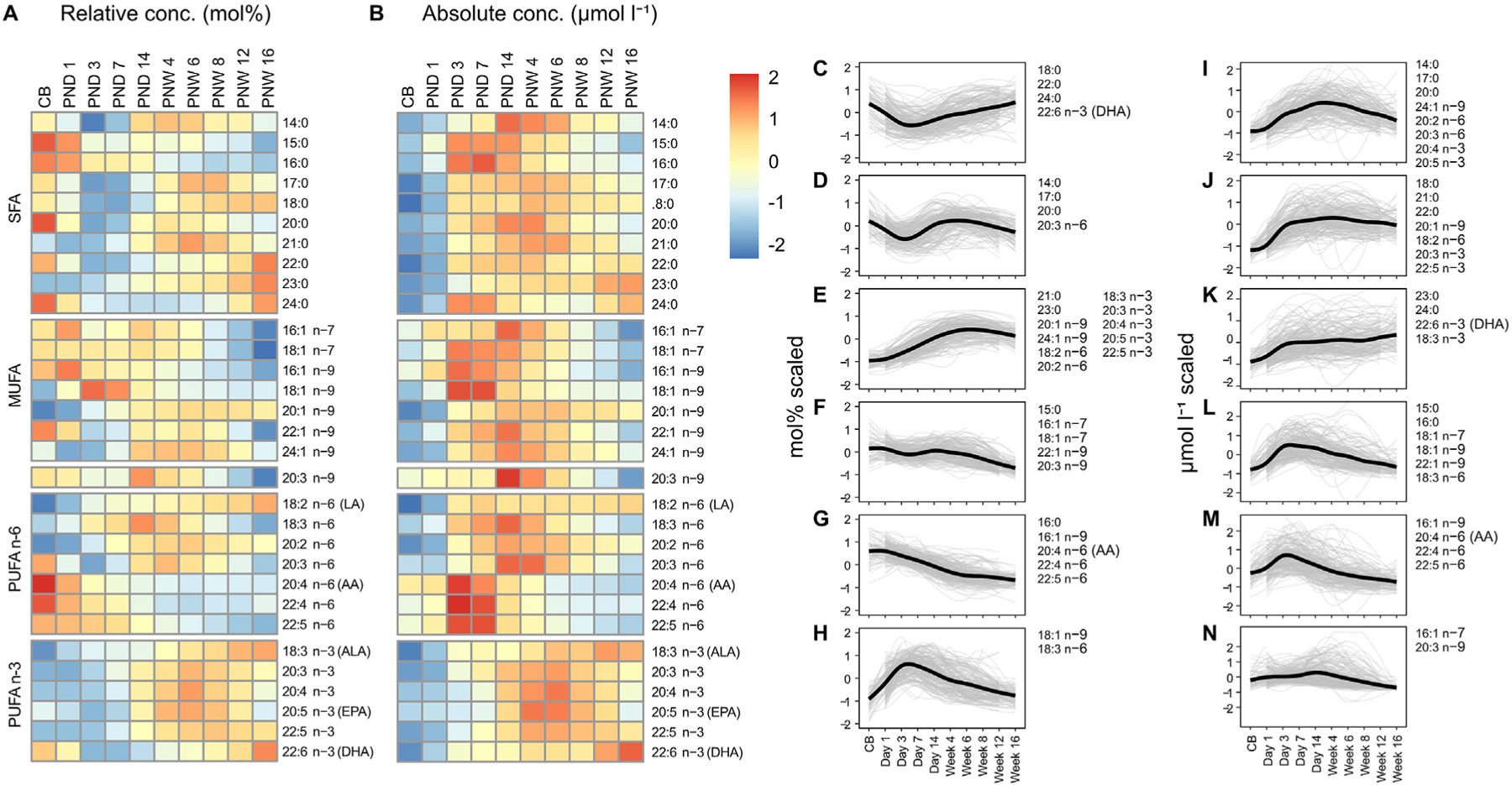
Infant serum fatty acid profiles. Heatmaps showing the mean serum levels of phospholipid fatty acids over the 16 first weeks of infant life based on relative (**A**) and absolute quantification (**B**). The levels of each fatty acid were subjected to Z-score normalization and colored accordingly to the legend. **C–H** and **I–N** show clusters of fatty acids with similar postnatal profiles based on mol% and μmol l^−1^, respectively. The thick black lines represent smoothed conditional means for all individuals (grey lines) and fatty acid indicated in the cluster. CB, cord blood; PND, postnatal days; PNW, postnatal weeks; SFA, saturated fatty acids; MUFA, monounsaturated fatty acids; PUFA n–3/n–6, polyunsaturated fatty acids omega-3/omega-6.

We visualized the fatty acid data for the cohort as a whole in heatmaps to further explore postnatal level changes. This showed prominent pattern differences when levels were expressed in relative (mol%, Fig. 4A) or absolute (μmol l^−1^, Fig. 4B) units. For example, the mean mol% of the n–6 fatty acids adrenic (22:4 n–6), docosapentaenoic (22:5 n–6), and AA displayed a successive decrease from cord blood to postnatal week 16 (Fig. 4A). However, the mean μmol l ^−1^ of these fatty acids increased from cord blood to day 3 and then declined over the study period (Fig. 4B).

Clustering the different fatty acids according to their postnatal level changes (Fig. S6) showed distinct patterns when expressed in relative or absolute units (Fig. 4C–N). For the data based on relative quantification, four out of six clusters showed decreased fatty acid levels after birth (Fig. 4C, D, F, and G), and two clusters showed increased levels (Fig. 4E and H). Many fatty acids clustered according to fatty acid family, i.e., the n–7 monounsaturates were found in one cluster (Fig. 4F), and the n–6 LCPUFAs grouped into two clusters (Fig. 4E and G) except for dihomo-γ-linolenic acid (20:3 n–6). Notably, DHA did not share profile with the other n–3 fatty acids.

Concerning the absolute quantification, no cluster showed a decrease after birth; five clusters included fatty acids that increased in concentration until at least postanatal day 3–7 (Fig. 4I–M), and one cluster comprised minor fatty acids with little detectable change until day 14 (Fig. 4N).

**Fig. 5.**
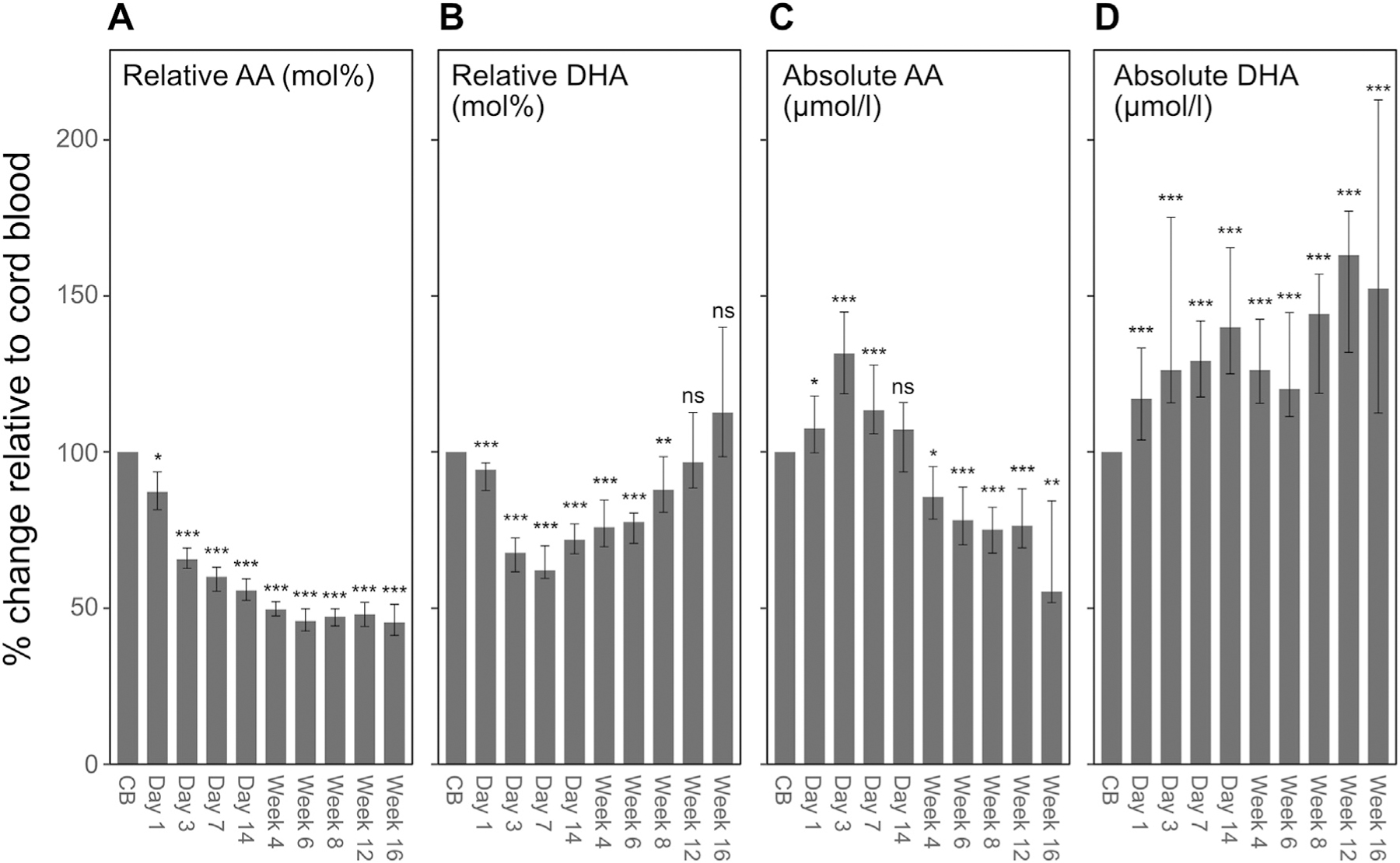
Changes in serum fatty acids relative to cord blood levels. Postnatal serum fatty acid normalized to concentrations in cord blood (n = 65). (**A**) mol% AA, (**B**) mol% DHA, (**C**) μmol l^−1^ AA, and (**D**) μmol l^−1^ DHA. Bars show medians and whiskers 95% CI. Asterisks denote statistically significant compared to cord blood (Wilcoxon signed-rank test, *p ≤ 0.05, **p ≤ 0.01; ***p ≤ 0.001; ns, nonsignificant).

To further explore differences between relative and absolute quantification, we calculated the percent change in AA and DHA from cord blood to postnatal time points (Fig. 5). This analysis included the 65 infants where cord blood samples were available. The mol% AA was higher in cord blood than at all later time points (p < 0.001, Fig. 5A). After an initial decline in the mol% DHA from birth to day 7, levels increased and were no longer significantly different from cord blood at postnatal week 12 (Fig. 5B). For the absolute levels, AA significantly increased to day 3 (p < 0.001), then declined, and was significantly lower compared to cord blood from week 4 (Fig. 5C). The absolute concentration of DHA increased after birth and was significantly (p < 0.001) higher at all postnatal time points compared to cord blood (Fig. 5D).

### 3.6. The inter-individual variation in fatty acid profiles changes with postnatal age

**Fig. 6.**
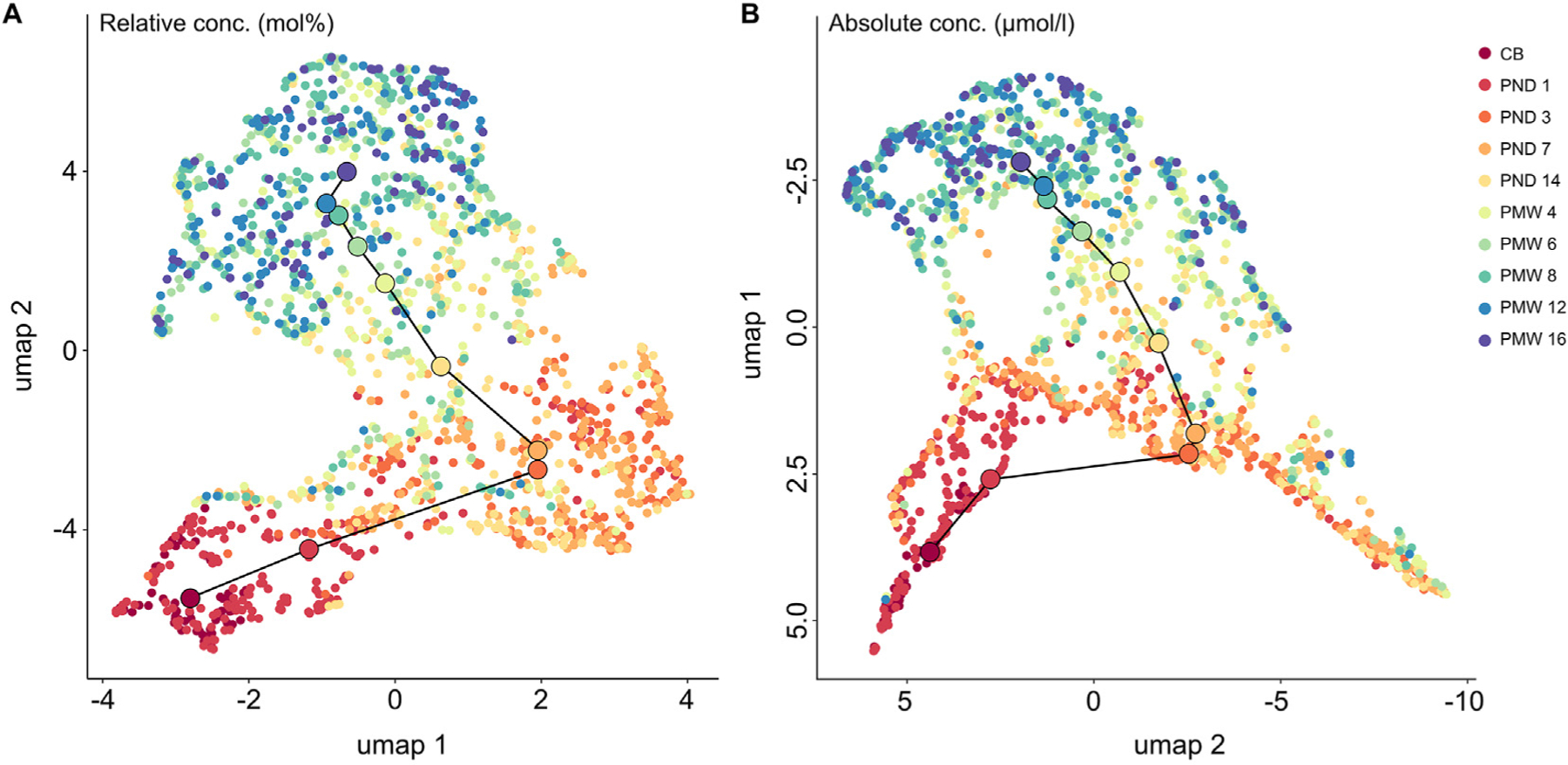
Uniform Manifold Approximation and Projection (UMAP) of all samples collected from birth to postnatal week 16 based on relative (**A**) and absolute quantification (**B**). Dot color represents the time of sampling according to legend. The larger dots represent means for each respective time point, and these have been connected to highlight common time-dependent changes in fatty acid profiles. CB, cord blood; PND, postnatal days; PNW, postnatal weeks.

We used Uniform Manifold Approximation and Projection (UMAP) to reduce the dimensionality of the data and compare individuals’ complete fatty acid profiles (Fig. 6). This analysis showed time-dependent sample clustering regardless if the data was expressed in relative (Fig. 6A) or absolute units (Fig. 6B).

Cord blood and day 1 samples clustered together while samples collected from postnatal day 3 to postnatal week 4 displayed higher heterogeneity; the latter was especially true for when the fatty acid content was expressed as absolute serum concentration. Samples from week 6 to week 12 formed a relatively cohesive cluster in both data sets (relative and absolute).

## 4. Discussion

This study provides a comprehensive overview of the extremely preterm infants’ fatty acid status during early postnatal life and how fatty acid levels are affected by external lipid sources. The largest changes in serum fatty acid levels occurred in the first few weeks after birth. This period was also defined by a sizeable inter-individual variability, which could partly be explained by the intake of parenteral lipids.

### 4.1. Effect of parenteral lipids on infant serum phospholipids and LCPUFAs

The strikingly different patterns observed for relative (mol%) and absolute (μmol l^−l^) fatty acid concentrations were related to a significant increase in total serum phospholipids starting soon after birth. The magnitude of this phospholipid expansion appears to be strongly related to parenteral lipid administration: at one week postnatal age, infants who received the most parenteral lipids (the ≥75th percentile category) showed around 40% higher total serum phospholipid fatty acids compared to infants who received the lowest amount of parenteral lipids (the ≤25th percentile category). Nevertheless, infants who primarily received enteral nutrition also showed an increase in total serum phospholipid fatty acids in the first week of life. Thus, the increase in total serum phospholipids appears to represent a physiological response to extrauterine life and the transition from glucose to fat as the main energy source. Previous studies have shown a similar increase in the concentration of serum/plasma phospholipids after birth in preterm infants [28–31].

Intravenous lipid emulsions and fat-soluble vitamin supplements are emulsified with egg yolk phospholipids that contain some DHA and AA [11,28]. We found that administration of parenteral lipids resulted in higher absolute infant DHA and AA at day 7, but lower relative levels of these LCPUFAs. As we sampled blood during continuous lipid infusion, it is clear that the phospholipid composition of the intravenous lipid emulsion impacted infant serum lipid composition. The main fatty acid of the intravenous lipid emulsion used in this trial is oleic acid (approximately 55% [17]). Accordingly, oleic acid was among the serum fatty acid that showed the largest postnatal concentration change, with a rapid but transient increase during the first two weeks of life.

As seen over the whole study period, infants on high parenteral lipids displayed lower relative levels of both DHA and AA. Robinson et al. showed that red blood cell (RBC) DHA, but not AA, decreased more in extremely low birth weight infants with prolonged exposure (>28 days) to a lipid emulsion based on soybean oil than in infants with shorter exposure (≤28 days) [32]. However, numerically, also RBC AA levels were lower in the group with longer exposure to the lipid emulsion. The addition of both DHA and AA to the intravenous lipid emulsion may help to ameliorate infant LCPUFA deficiency.

### 4.2. Longitudinal AA and DHA and the impact of enteral supplementation

In preterm infant blood, AA and DHA are mainly transported as esters of phospholipids in lipoproteins that supply tissues with the material needed for metabolism and cell expansion [31]. We found that the absolute phospholipid DHA concentration increased after birth and was higher at all postnatal time points (up to 16 weeks) compared to that in cord blood, indicating that DHA available for accretion is maintained in the postnatal period. However, although the absolute concentration of AA initially increased, at week 4 and beyond the serum AA concentration was lower compared to that in cord blood. The accumulation of AA in the forebrain is markedly higher than that of DHA during the third trimester *in utero* [39]. We speculate that the decline seen in serum AA reflects the high demand of the developing brain and other tissues. Our data suggest that further efforts are needed to increase preterm infant AA and the availability of this important LCPUFA for accretion.

As we reported previously [12], the AA:DHA supplement increased the relative proportions of infant serum AA and DHA. Here, we further show that the supplementation resulted in a significant increase in absolute AA levels and a trend towards increased absolute DHA levels. Concerns have been raised that fish oil supplementation increases infant blood EPA to unphysiological levels, and skews the n–3:n–6 LCPUFA ratio [33]. In our study the intervention had minimal effects on fatty acids other than AA and DHA, demonstrating that the lipid supplementation strategy could precisely modulate the target fatty acids.

Collins et al. investigated dose–response of emulsified DHA via tube feeding on circulating RBC levels in infants born less than 30 weeks’ GA [34]. Administration of 120 mg/kg/day DHA during the first four weeks of life increased the molar fraction of DHA in RBC phospholipids by nearly 50%. In the N3RO trial (N-3 fatty acids for improvement in Respiratory Outcomes) of infants born before 29 weeks GA, emulsified DHA at a daily dose of 60 mg/kg significantly increased the whole blood level of DHA (3.9 weight% vs 2.5 weight% in the control group at mean 36.1 weeks GA). In comparison, we report a modest increase in infant serum phospholipid AA and DHA, although the fatty acids were given at a relatively high dose, 100 and 50 mg/kg daily, respectively. The recent ImNuT (Immature, Nutrition Therapy) trial, also enterally supplementing preterm infants with AA:DHA at 100:50 mg/kg/day, reported a small increase in the molar percent of whole blood DHA between day 2 and PMA 36 weeks but no significant effect on AA levels [20]. In both this and the ImNuT trial, the supplement was provided as pure oil, and emulsification of the fat would likely have enhanced the LCPUFA bioavailability, as demonstrated in both experimental and clinical studies [35,36]. Increased supplement dosing and/or improved lipid delivery may be needed to avoid infant LCPUFA shortfall.

### 4.3. Fatty acid clustering

We grouped fatty acids according to postnatal level profiles and found several distinct clusters. Some fatty acids clustered according to class; for example, the proportion of the n–3 fatty acids, except DHA, were found in one cluster. The proportion of DHA, on the other hand, shared profile with the long-chain saturates stearic (18:0), behenic (22:0), and lignoceric acid (24:0). The n–6 LCPUFAs AA, adrenic (22:4 n–6), and docosapentaenoic acid (22:5 n–6) clustered together in data based on both relative and absolute concentration, albeit with very different profiles. This analysis highlights the complexity of lipid metabolism during early postnatal life and calls for further studies to determine the origin and fate of circulatory fatty acids.

### 4.4. Strengths and limitations

There are limitations to our study. First, infants in the control group did not receive a placebo treatment. However, there was no difference in total lipid intake between the active and the control group. Second, when investigating the impact of parenteral lipids on infant LCPUFA levels we did not account for other potential confounders such as blood transfusions, LCPUFAs in human milk, LCPUFAs in the human milk fortifiers, and lipids present in other fortifiers, which may impact infant plasma levels [37,38]. Third, we used a postnatal timescale when assessing fatty acid levels, although sample collection was based on both postnatal age and postmenstrual age. As a result, infants born at the lowest GAs were overrepresented in numbers at later time points. However, for statistical analyses, we limited the time so that there was a similar distribution of the three GA strata. The strengths of this study lie in the high temporal resolution, the relatively large number of infants recruited at several centers, carefully weighted GA groups, prospectively daily collected nutritional data, and the combined reporting of fatty acids levels in relative and absolute units.

### 4.5. Conclusions

Following preterm birth, infant LCPUFA levels fall below those of the fetus *in utero*. The long-term consequences of this LCPUFA deprivation during a sensitive time of development are not yet fully understood. No current nutritional management program restores preterm infant fatty acid profiles to match the intrauterine situation, and whether this is achievable or even desirable, has not been established. This and other studies have provided in-depth knowledge on circulating fatty acids in the preterm infant in the first months of life. However, there is limited consensus on the ideal fatty acid supplementation and profile to aim for when feeding the preterm infant to promote optimal growth and long-term health, warranting further studies.

## Supplementary Material

supplementary material

## Figures and Tables

**Table 1:** Infant characteristics.

Variable	All (n = 204)	Control (n = 103)	AA:DHA (n = 101)	p-value control v AA:DHA
Gestational age (weeks)	25.5 (1.4)	25.5 (1.4)	25.5 (1.5)	0.97
	25.5 (22.3; 27.9)	25.6 (22.9; 27.9)	25.4 (22.3; 27.9)	
Birth weight (g)	789 (196)	782 (196)	797 (197)	0.59
	770 (411; 1345)	764 (411; 1330)	775 (455; 1345)	
Birth weight SDS	0.07 (0.83)	0.01 (0.84)	0.13 (0.81)	0.28
	0.13 (−2.30; 2.34)	0.13 (−2.30; 1.81)	0.24 (−2.25; 2.34)	
Female	87 (42.6)	44 (42.7)	43 (42.6)	1.00
Death	27 (13.2)	10 (9.7)	17 (16.8)	0.15

SDS = standard deviation score.

For categorical variables n (%) is presented.

For continuous variables Mean (SD)/Median (Min; Max) is presented.

**Table 2:** Birth characteristics of infants grouped by intake of parenteral lipids in the first four weeks of life. Percentages represent the fraction of infants per parenteral lipid group (column-wise).

Variable	≤25th percentile (n = 48)	25th-75th percentile (n = 102)	≥75th percentile (n = 53)	p-value^b^
Gestational age at birth (weeks)	26.3 (1.2)	25.4 (1.4)	25.0 (1.3)	<0.0001
	26.4 (23.1; 27.9)	25.3 (22.6; 27.9)	25 (22.3; 27.3)	
Female	22 (45.8%)	45 (44.1%)	20 (37.7%)	0.41
Birth weight (g)	914 (192)	770 (193)	714 (154)	<0.0001
	890 (550; 1345)	728 (420; 1220)	698 (411; 1160)	
Birth weight SDS	0.31 (0.68)	0.03 (0.87)	−0.10 (0.84)	0.020
	0.29 (−1.33; 2.34)	0.13 (−2.29; 1.61)	−0.07 (−2.30; 1.57)	
Center				
1	22 (45.8%)	36 (35.3%)	14 (26.4%)	
2	5 (10.4%)	28 (27.5%)	31 (58.5%)	
3^a^	21 (43.8%)	38 (37.3%)	8 (15.1%)	<0.0001
Randomized treatment group				
Control	21 (43.8%)	55 (53.9%)	27 (50.9%)	
AA:DHA	27 (56.3%)	47 (46.1%)	26 (49.1%)	0.49

SDS = standard deviation score.

For categorical variables n (%) is presented.

For continuous variables Mean (SD)/Mesdian (Min; Max) is presented.

a One infant had missing nutrition data at center 3. Therefore, 203 patients are analyzed in total in this table.

b Mantel-Haenszel Chi-Square test for dichotomous variables and Jonckheere-Terpstra test for continuous variables.
