# Supplementary material for: Modification of serum fatty acids in preterm infants by parenteral lipids and enteral docosahexaenoic acid/arachidonic acid: A secondary analysis of the Mega Donna Mega trial
Source: Clin Nutr. Author manuscript; Available in PMC 2023 Sep 21. (PMC10512593; doi:10.1016/j.clnu.2023.04.020)
Supplement: supplementary material [file NIHMS1932044-supplement-supplementary_material.pdf]

Supplemental Material to

Modification of serum fatty acids in preterm infants by parenteral lipids and enteral docosahexaenoic acid/arachidonic acid: A secondary analysis of the Mega Donna Mega trial

Ulrika Sjöbom, Mats X Andersson, Aldina Pivodic, Anna-My Lund, Mireille Vanpee, Ingrid Hansen-Pupp, David Ley, Dirk Wackernagel, Karin Sävman, Lois EH Smith, Chatarina Löfqvist, Ann Hellström, and Anders K. Nilsson (anders.k.nilsson@gu.se)

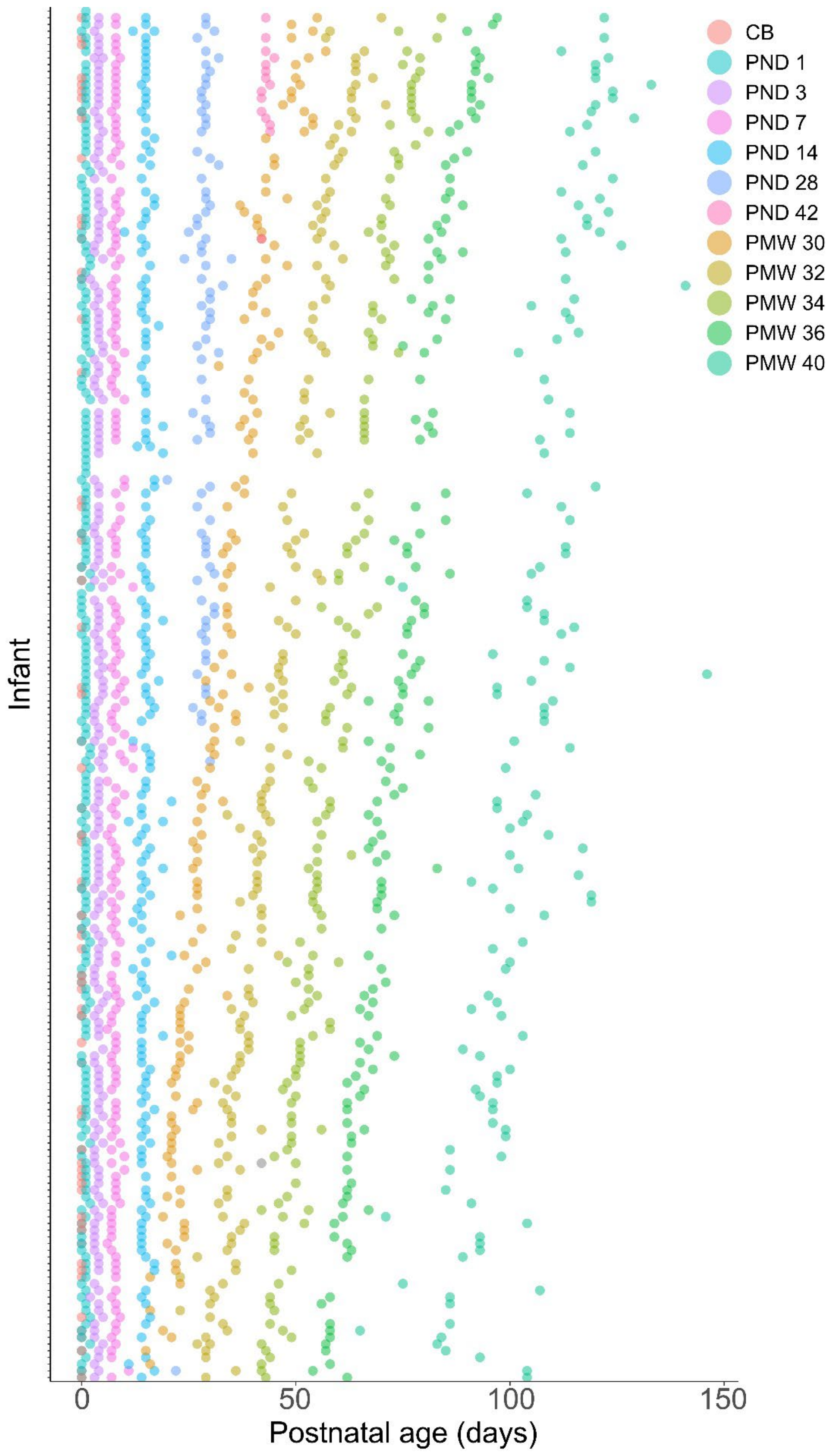

Fig S1. Samples analyzed in this study colored by time of sampling according to the study protocol. Each row in the figure represents one infant and each dot a fatty acid sample. Infants have been ordered by gestational age at birth. PND, postnatal day; PMW, postmenstrual week.

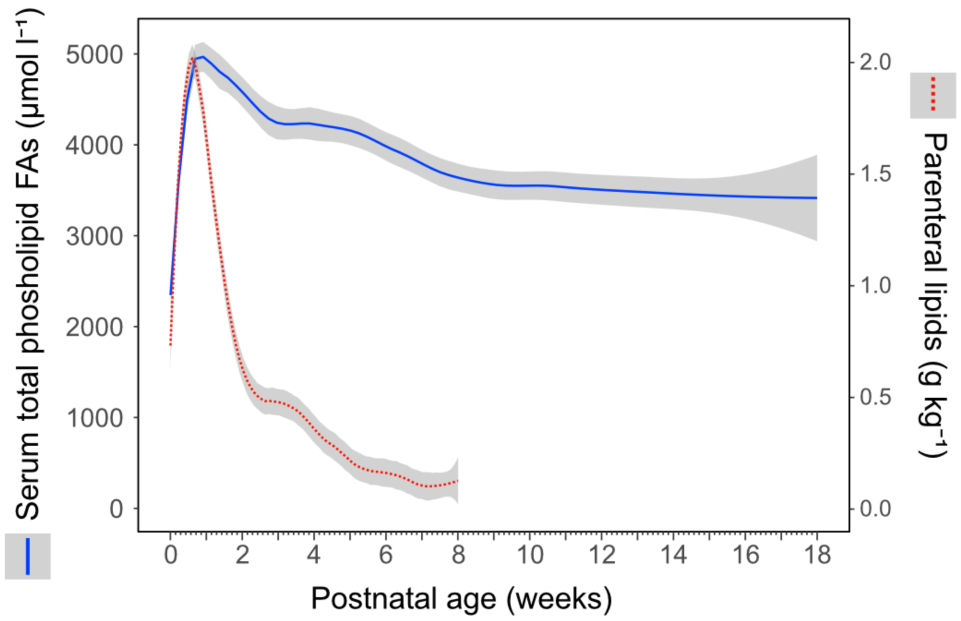

Figure S2. Total serum phospholipid fatty acids (solid blue line and left y-axis) and daily parenteral lipid administration (dotted red line and right y-axis). Lines represent smoothed conditional means with 95% confidence intervals (shaded areas).

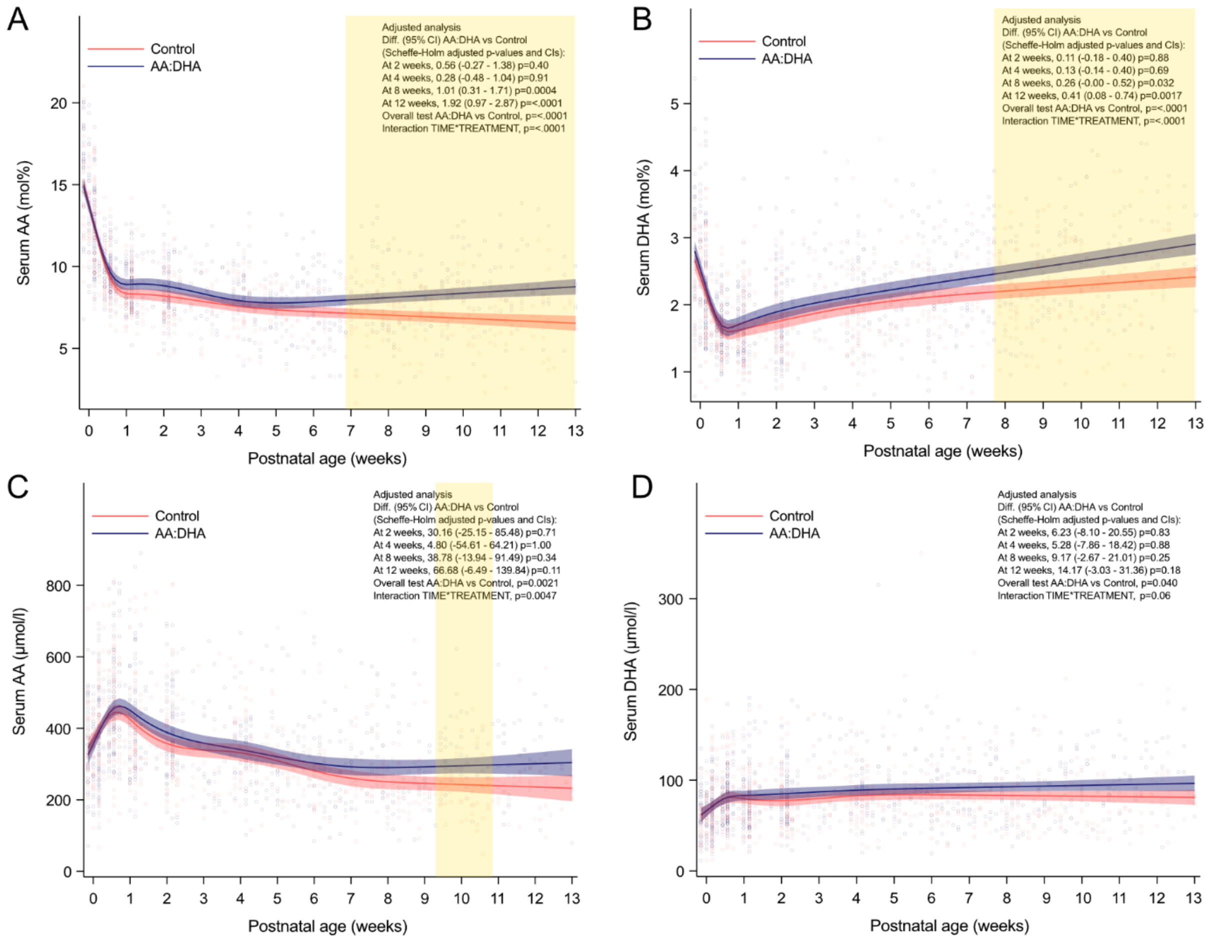

Figure S3. Serum phospholipid levels of AA and DHA according to intervention group.

Relative (mol%) serum phospholipid concentration of AA (**A**) and DHA (**B**). Absolute (µmol l^{-1}) serum phospholipid concentration of AA (**C**) and DHA (**D**). Scatters show actual measured values and lines the estimates (with 95% CI) from mixed models for repeated measures adjusted for GA at birth, center, birth weight, all three variables including interaction with gestational age (TIME) and sex. Graph area highlighted in yellow represents periods where curves differ significantly between AA:DHA and Control group after adjustment for multiplicity. N=103 for control and n=101 for AA:DHA.

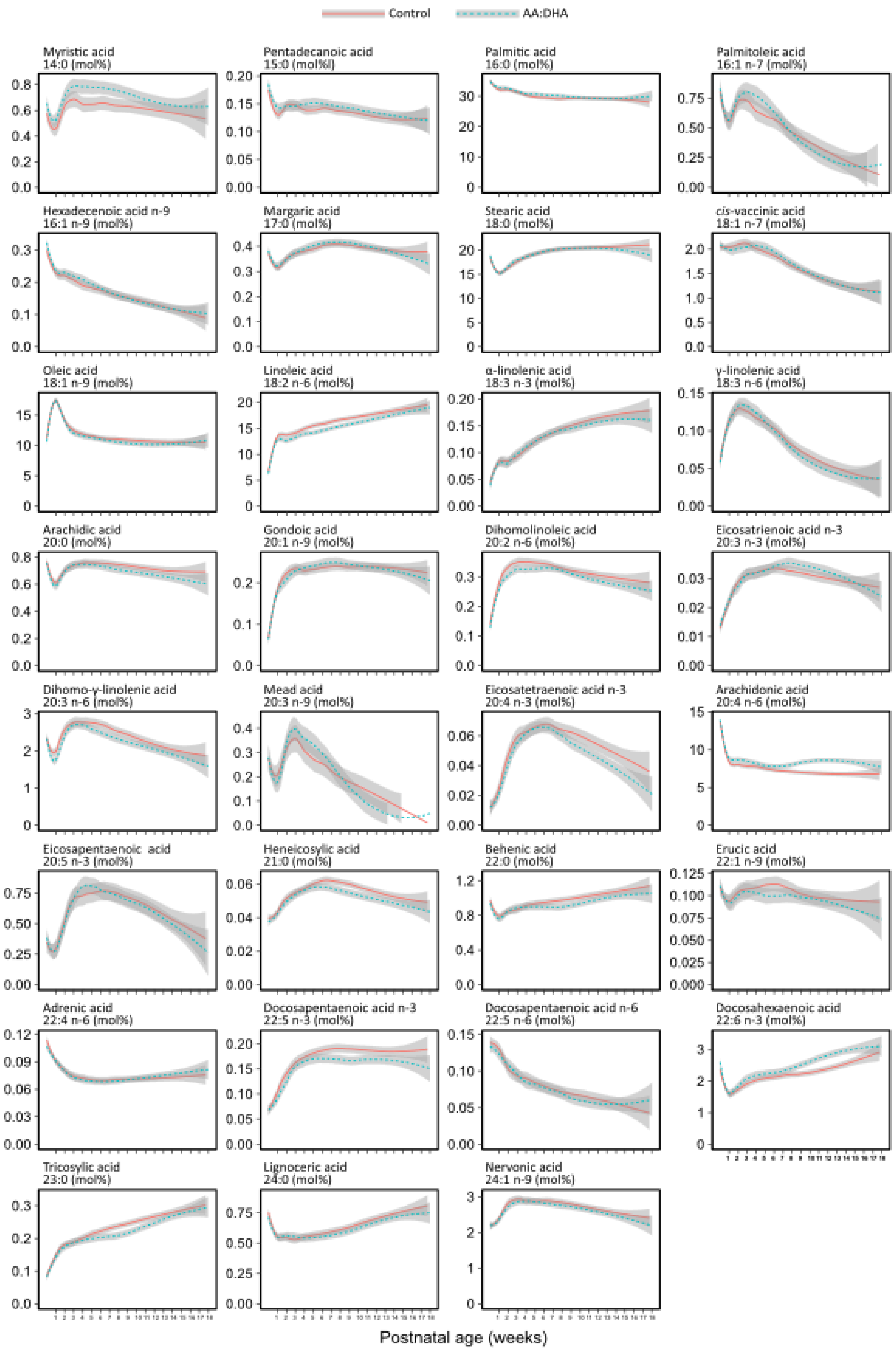

Figure S4. Relative (mol%) infant serum levels of all quantified fatty acids in this study according to intervention group (solid red line Control, dashed blue line AA:DHA). Lines represent smoothed conditional means with 95% confidence intervals (shaded areas).

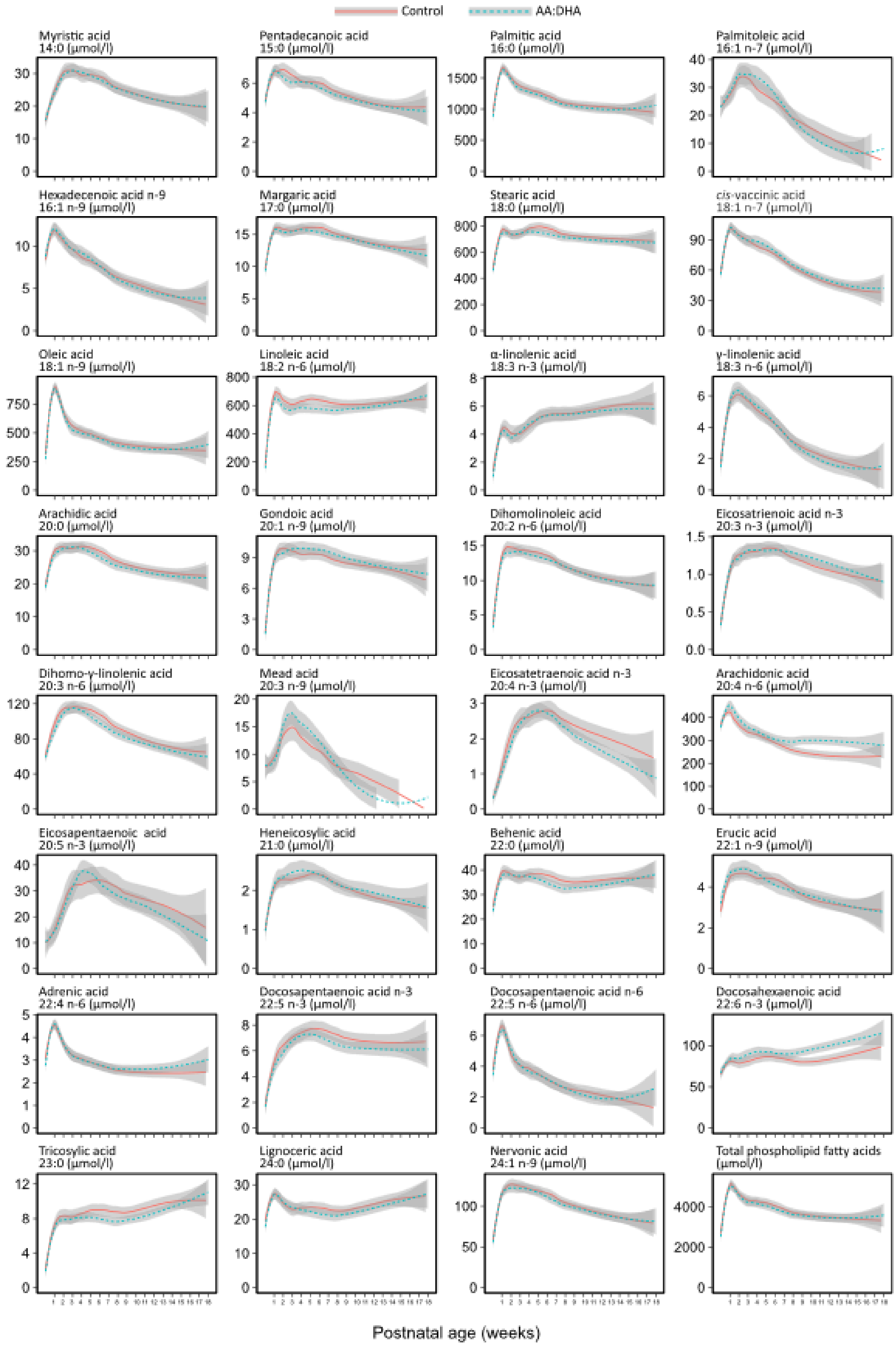

Figure S5. Absolute (μmol l^{-1}) infant serum levels of all quantified fatty acids in this study according to intervention group (solid red line Control, dashed blue line AA:DHA). Lines represent smoothed conditional means with 95% confidence intervals (shaded areas).

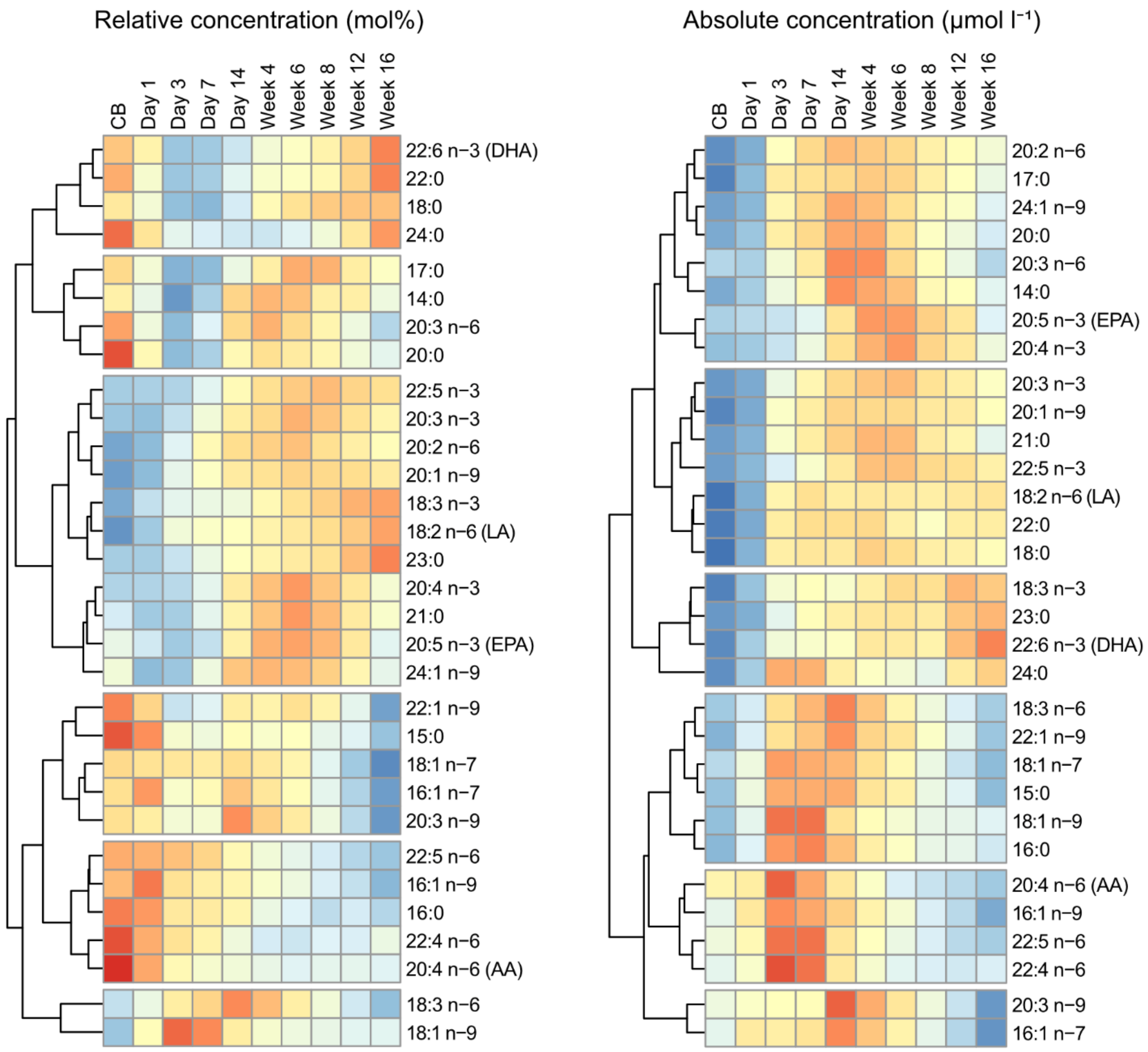

Figure S6. Hierarchical clustering of fatty acid patterns based on relative (left panel) and absolute (right panel) quantification.

Table S1. Fluid, human milk, and lipid intake during postnatal days 1-28. Only infants who survived the first 28 postnatal days are included. Shown are medians (Q1-Q3) of mean daily intake.

	Variable	Control (n=98)	AA:DHA (n=89)	p-value*
Week 1	Fluids total (ml/kg/d)	140.4 (130.9-150.4)	139.3 (129.9-151.2)	0.56
	Fluids parenteral (ml/kg/d)	81.4 (64.1-111.1)	85.5 (57.1-116.4)	0.86
	Human milk (ml/kg/d)	58 (37.1-69.2)	51.6 (33.7-71.6)	0.67
	Lipids total (g/kg/d)	3.9 (3.4-4.3)	3.9 (3.5-4.5)	0.20
	Lipids enteral (g/kg/d)	2.1 (1.3-2.7)	2.2 (1.6-3)	0.16
	Lipids parenteral (g/kg/d)	1.7 (1.3-2.3)	1.6 (1.1-2)	0.19
Week 2	Fluids total (ml/kg/d)	166.3 (160.7-174)	169.1 (160.1-179.4)	0.11
	Fluids parenteral (ml/kg/d)	36.5 (6.1-72.2)	38.6 (7.7-82.6)	0.84
	Human milk (ml/kg/d)	123.8 (92-154.5)	123.4 (93.2-155.4)	0.82
	Lipids total (g/kg/d)	5.8 (4.7-6.7)	5.8 (5-7)	0.57
	Lipids enteral (g/kg/d)	5.1 (3.3-6.4)	5 (3.5-6.7)	0.63
	Lipids parenteral (g/kg/d)	0.7 (0-1.7)	0.6 (0-1.5)	0.98
Week 3	Fluids total (ml/kg/d)	167.8 (159.7-176)	171.5 (163.9-178.2)	0.06
	Fluids parenteral (ml/kg/d)	17 (0-54.5)	9.6 (0.1-37.1)	0.59
	Human milk (ml/kg/d)	144.6 (108-165.9)	154.6 (123.2-168.8)	0.13
	Lipids total (g/kg/d)	6.7 (5.3-7.7)	6.6 (5.6-7.9)	0.90
	Lipids enteral (g/kg/d)	6.6 (4.8-7.6)	6.5 (5.2-7.7)	0.72
	Lipids parenteral (g/kg/d)	0 (0-0.5)	0 (0-0.3)	0.63
Week 4	Fluids total (ml/kg/d)	167.2 (160.3-174.2)	169.1 (162.3-177.1)	0.28
	Fluids parenteral (ml/kg/d)	3.1 (0-38.4)	1.1 (0-22.5)	0.58
	Human milk (ml/kg/d)	154.3 (123-164.6)	153.6 (137.1-167)	0.54
	Lipids total (g/kg/d)	7.1 (5.8-8)	6.9 (6-8.1)	0.99
	Lipids enteral (g/kg/d)	7.1 (5.1-7.9)	6.9 (5.8-8.1)	0.88
	Lipids parenteral (g/kg/d)	0 (0-0.3)	0 (0-0)	0.07
Week 1-4	Fluids total (ml/kg/d)	161 (155.4-166.5)	163.2 (157.5-168.5)	0.14
	Fluids parenteral (ml/kg/d)	44.1 (21.5-68)	40 (20.2-69.5)	0.54
	Human milk (ml/kg/d)	114.8 (89.2-134.7)	119.4 (93.7-137.7)	0.36
	Lipids total (g/kg/d)	5.7 (5.1-6.5)	5.9 (5.1-6.6)	0.58
	Lipids enteral (g/kg/d)	5 (3.9-5.9)	5.2 (4-6.3)	0.34
	Lipids parenteral (g/kg/d)	0.7 (0.4-1.4)	0.6 (0.3-1.1)	0.24

* Mann-Whitney U test
